# Supplementary figures and images for: Synchronised provisioning at the nest: parental coordination over care in a socially monogamous species
Source: PeerJ. 2013 Dec 19;1:e232. doi: 10.7717/peerj.232 (PMC3883492; doi:10.7717/peerj.232)

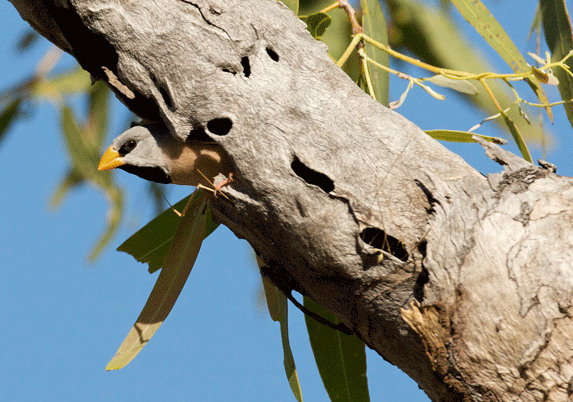

Supplement: Supplemental Information 1 — Adult long-tailed finch emerging from nest. [file peerj-01-232-s001.jpg]
